# Supplementary material for: Improving Data-Informed Care in New Brunswick Long-Term Care Homes: A Qualitative Study on an Educational Intervention for interRAI Coordinators
Source: Healthcare (Basel). 2024 Dec 23;12(24):2592. doi: 10.3390/healthcare12242592 (PMC11727779; doi:10.3390/healthcare12242592)
Supplement: Supplementary file 1 [file healthcare-12-02592-s001.zip › healthcare-3379480-supplementary.pdf]

Improving data-informed care in New Brunswick long-term care homes:
A qualitative study on and educational intervention for interRAI coordinators

Supplementary materials

Standards for Reporting Qualitative Research (SRQR)*

http://www.equator-network.org/reporting-guidelines/srqr/

Page/line no(s).

Title and abstract

**Title** - Concise description of the nature and topic of the study Identifying the study as qualitative or indicating the approach (e.g., ethnography, grounded theory) or data collection methods (e.g., interview, focus group) is recommended	1
**Abstract** - Summary of key elements of the study using the abstract format of the intended publication; typically includes background, purpose, methods, results, and conclusions	1

Introduction

**Problem formulation** - Description and significance of the problem/phenomenon studied; review of relevant theory and empirical work; problem statement	1-3
**Purpose or research questio**n - Purpose of the study and specific objectives or questions	3

Methods

**Qualitative approach and research paradigm** - Qualitative approach (e.g., ethnography, grounded theory, case study, phenomenology, narrative research) and guiding theory if appropriate; identifying the research paradigm (e.g., postpositivist, constructivist/ interpretivist) is also recommended; rationale**	4
**Researcher characteristics and reflexivity** - Researchers' characteristics that may influence the research, including personal attributes, qualifications/experience, relationship with participants, assumptions, and/or presuppositions; potential or actual interaction between researchers' characteristics and the research questions, approach, methods, results, and/or transferability	4
**Context** - Setting/site and salient contextual factors; rationale**	4
**Sampling strategy** - How and why research participants, documents, or events were selected; criteria for deciding when no further sampling was necessary (e.g., sampling saturation); rationale**	3-4
**Ethical issues pertaining to human subjects** - Documentation of approval by an appropriate ethics review board and participant consent, or explanation for lack thereof; other confidentiality and data security issues	3

**Data collection methods** - Types of data collected; details of data collection procedures including (as appropriate) start and stop dates of data collection and analysis, iterative process, triangulation of sources/methods, and modification of procedures in response to evolving study findings; rationale**	4
**Data collection instruments and technologies** - Description of instruments (e.g., interview guides, questionnaires) and devices (e.g., audio recorders) used for data collection; if/how the instrument(s) changed over the course of the study	4
**Units of study** - Number and relevant characteristics of participants, documents, or events included in the study; level of participation (could be reported in results)	5
**Data processing** - Methods for processing data prior to and during analysis, including transcription, data entry, data management and security, verification of data integrity, data coding, and anonymization/de-identification of excerpts	4
**Data analysis** - Process by which inferences, themes, etc., were identified and developed, including the researchers involved in data analysis; usually references a specific paradigm or approach; rationale**	4
**Techniques to enhance trustworthiness** - Techniques to enhance trustworthiness and credibility of data analysis (e.g., member checking, audit trail, triangulation); rationale**	4

Results/findings

**Synthesis and interpretation** - Main findings (e.g., interpretations, inferences, and themes); might include development of a theory or model, or integration with prior research or theory	5-11
**Links to empirical data** - Evidence (e.g., quotes, field notes, text excerpts, photographs) to substantiate analytic findings	5-11

Discussion

**Integration with prior work, implications, transferability, and contribution(s) to the field -** Short summary of main findings; explanation of how findings and conclusions connect to, support, elaborate on, or challenge conclusions of earlier scholarship; discussion of scope of application/generalizability; identification of unique contribution(s) to scholarship in a discipline or field	11
**Limitations** - Trustworthiness and limitations of findings	11

Other

**Conflicts of interest** - Potential sources of influence or perceived influence on study conduct and conclusions; how these were managed	12
**Funding** - Sources of funding and other support; role of funders in data collection, interpretation, and reporting	11

Improving data-informed care in New Brunswick long-term care homes:
A qualitative study on and educational intervention for interRAI coordinators

*The authors created the SRQR by searching the literature to identify guidelines, reporting standards, and critical appraisal criteria for qualitative research; reviewing the reference lists of retrieved sources; and contacting experts to gain feedback. The SRQR aims to improve the transparency of all aspects of qualitative research by providing clear standards for reporting qualitative research.

**The rationale should briefly discuss the justification for choosing that theory, approach, method, or technique rather than other options available, the assumptions and limitations implicit in those choices, and how those choices influence study conclusions and transferability. As appropriate, the rationale for several items might be discussed together.

Reference:
O'Brien BC, Harris IB, Beckman TJ, Reed DA, Cook DA. Standards for reporting qualitative research: a synthesis of recommendations. Academic Medicine, Vol. 89, No. 9 / Sept 2014
DOI: 10.1097/ACM.0000000000000388

Semi-structured interview guide:

1 Can you tell me a little bit about your role in the nursing home?

2 How did you find the education sessions?

3 Was there anything that took you by surprise during the sessions?
   i. Prompt: using the interRAI system, NB Quality Indicators

4 Is there anything you didn't like about those education sessions?

5 Did you feel comfortable offering your opinions and ideas during the sessions?
   i. Follow-up: Did you feel your questions were answered appropriately whenever you asked them?

6 Has your comfort with accessing interRAI data changed since you attended the sessions?

7 What were your initial reactions to hearing the interRAI data and information in NB residents?
   Probe: surprised, worried, angry, happy, motivated, disappointed

8 What quality indicators were you interested in hearing about at the sessions?
   i. Follow-up: Did you find they were adequatel addressed in the sessions?

9 What potential solutions have you be heard of or have you been using for QI with the interRAI data?

10 What would you like to see for next steps using this data in your home and across New Brunswick?

11 Is there anything that you think should be done differently in the education sessions?

   i. Follow-up: Would you recommend the sessions for other nursing staff?

12 Have you used anything you learned from those sessions to change your quality improvement plan?

13 Is there anything else you would like to say about InterRAI or the sessions that you attended on the interRAI or anything else you would like to suggest or comment or critique?
